# Molecular Mechanisms Responsible for the Therapeutic Potential of Mesenchymal Stem Cell-Derived Exosomes in the Treatment of Lung Fibrosis

**DOI:** 10.3390/ijms25084378

**Published:** 2024-04-16

**Authors:** Carl Randall Harrell, Valentin Djonov, Ana Volarevic, Aleksandar Arsenijevic, Vladislav Volarevic

**Affiliations:** 1 Regenerative Processing Plant, LLC, 34176 US Highway 19 N, Palm Harbor, FL 34684, USA; dr.harrell@regenerativeplant.org; 2 Institute of Anatomy, University of Bern, Baltzerstrasse 2, 3012 Bern, Switzerland; valentin.djonov@unibe.ch; 3 Department of Psychology, Center for Research on Harmful Effects of Biological and Chemical Hazards, Faculty of Medical Sciences, University of Kragujevac, 69 Svetozara Markovica Street, 34000 Kragujevac, Serbia; ana.volarevic@medf.kg.ac.rs; 4 Departments of Genetics, Microbiology and Immunology, Center for Research on Harmful Effects of Biological and Chemical Hazards, Faculty of Medical Sciences, University of Kragujevac, 69 Svetozara Markovica Street, 34000 Kragujevac, Serbia; 5 Faculty of Pharmacy Novi Sad, Trg Mladenaca 5, 21000 Novi Sad, Serbia

**Keywords:** mesenchymal stem cells, exosomes, lung fibrosis, immunomodulation, therapy

## Abstract

Mesenchymal stem cell-derived exosomes (MSC-Exos) are nano-sized extracellular vesicles which contain various MSC-sourced anti-fibrotic, immunoregulatory and angio-modulatory proteins (growth factors, immunoregulatory cytokines, chemokines), lipids, and nucleic acids (messenger RNA and microRNAs). Due to their lipid envelope, MSC-Exos easily by-pass all barriers in the body and deliver their cargo directly in target cells, modulating their viability, proliferation, phenotype and function. The results obtained in recently published experimental studies demonstrated beneficial effects of MSC-Exos in the treatment of lung fibrosis. MSC-Exos reduced activation of fibroblasts and prevented their differentiation in myofibroblasts. By delivering MSC-sourced immunoregulatory factors in lung-infiltrated monocytes and T cells, MSC-Exos modulate their function, alleviating on-going inflammation and fibrosis. MSC-Exos may also serve as vehicles for the target delivery of anti-fibrotic and immunomodulatory agents, enabling enhanced attenuation of lung fibrosis. Although numerous pre-clinical studies have demonstrated the therapeutic potential of MSC-Exos in the treatment of pulmonary fibrosis, there are several challenges that currently hinder their clinical implementation. Therefore, in this review article, we summarized current knowledge and we discussed future perspectives regarding molecular and cellular mechanisms which were responsible for the anti-fibrotic, anti-inflammatory and immunoregulatory properties of MSC-Exos, paving the way for their clinical use in the treatment of lung fibrosis.

## 1. Introduction

Lung or pulmonary fibrosis is a chronic and progressive lung disease characterized by the formation of excessive scar tissue, fibrosis, in the lungs [1]. This scarring occurs due to the abnormal accumulation of collagen and other extracellular matrix components, which leads to the thickening and stiffening of lung parenchymal [1,2]. The pathological process begins with an initial insult or injury to the lung, which can be caused by various factors such as environmental toxins, infections, autoimmune reaction or radiation exposure [2,3]. Alarmins and damage-associated molecular patterns (DAMPs), released from injured cells, bind to their receptors on the membrane of lung-infiltrated immune cells, leading to their activation [4]. Upon activation, neutrophils, alveolar macrophages and T-lymphocytes release pro-inflammatory cytokines, such as transforming growth factor-beta (TGF-β), tumor necrosis factor-alpha (TNF-α) and interleukins (IL)-1, IL-6, IL-13), which act as signaling molecules and, together with macrophage-sourced pro-fibrotic proteins (platelet-derived growth factor (PDGF) and fibroblast growth factor (FGF)), directly stimulate fibroblasts to produce collagen and extracellular matrix components [5]. Additionally, under the influence of TNF-α and TGF-β, alveolar epithelial cells (AECs) undergo transformation into mesenchymal-like cells with fibroblast-like characteristics, paving the way for the development and progression of lung fibrosis [6]. Upon DAMPs-dependent activation, alveolar macrophages produce TNF-α and IL-1β (which induce enhanced expression of E and P selectins on lung endothelial cells (ECs)) and neutrophil-, monocyte- and T cell-attracting chemokines (CCL2, CCL3 and CXCL12) which bind to their respective receptors on circulating leukocytes, enabling their massive influx in the fibrotic areas [4,5]. As a result, the lungs become infiltrated with immune cells, less elastic and unable to properly expand [1,2].

The main clinical symptoms and signs of lung fibrosis can vary depending on the stage and severity of the disease [7]. Dyspnea, dry cough, fatigue, chest pain and discomfort are the hallmark symptoms of lung fibrosis, which can be result of the underlying inflammation and scarring in the lung tissue [7]. Some patients may develop rapid, shallow breathing patterns (tachypnea) as they struggle to get enough oxygen [8]. As lung fibrosis progresses, the scarring and stiffening of lung tissue can severely impair the exchange of oxygen and carbon dioxide, which can result in life-threatening respiratory failure [9]. Lung fibrosis increases the risk of blood clots forming in the pulmonary arteries and can cause increased resistance to blood flow in the lungs, resulting in the development of pulmonary hypertension which further worsens respiratory symptoms and can lead to heart failure [10].

The treatment of lung fibrosis aims to slow down disease progression, manage symptoms and improve quality of life [11,12]. Anti-inflammatory and immunosuppressive drugs like corticosteroids, pirfenidone and nintedanib may be used to inhibit detrimental immune response and to slow down inflammation-driven pathological changes and fibrosis in the lungs. Supplemental oxygen therapy is often prescribed to individuals with lung fibrosis who have low oxygen levels in their blood [13]. For patients suffering from advanced lung fibrosis and severe respiratory impairment, lung transplantation represents the only possible therapeutic option [14]. However, the limited availability of suitable donor lungs and lifelong commitment to post-transplant immunosuppressive treatment limit the feasibility of lung transplantation. Despite immunosuppressant medications, the body’s immune system may still recognize the transplanted lung as foreign and mount an allogeneic immune response, leading to organ rejection [14,15]. Immunosuppressive drugs down-regulate the production of cytokines, which regulate the proliferation and activation of immune cells and attenuate immune cell-dependent protection against microbial pathogens [16]. When the immune system is weakened, harmful bacteria, viruses and fungi are able to grow extensively in the inflamed lungs [17]. Accordingly, continuous use of immunosuppressant medications can increase the risk of bacterial, viral or fungal pneumonia which can be severe and potentially life-threatening for lung transplant recipients [13,16]. Therefore, there is an urgent need for the therapeutic use of new anti-fibrotic and immunomodulatory agents which will be able to concurrently attenuate on-going lung inflammation and fibrosis without impairing protective immune response in the lungs of patients suffering from severe lung fibrosis [11,14].

## 2. Therapeutic Potential of Mesenchymal Stem Cells-Derived Exosomes in Pulmonary Fibrosis

Results recently obtained in large number of experimental studies indicated that mesenchymal stem cells (MSCs), self-renewable, adult, rapidly proliferative, multipotent cells, should be considered as potentially new therapeutic agents for the treatment of lung fibrosis [18,19,20,21]. MSCs produce various anti-inflammatory and immunosuppressive factors which may suppress immune cell-driven lung injury and inflammation, attenuate collagen production, inhibit epithelial to mesenchymal transition (EMT) and prevent tissue remodeling in the fibrotic lungs [20,21,22]. MSCs release hepatocyte growth factor (HGF) which binds to c-Met receptor on activated fibroblasts, inducing activation of the PI3K/Akt and Ras/MAPK pathways, which lead to the inhibition of fibroblast activation and the subsequent reduction in collagen synthesis [23]. Additionally, MSC-sourced HGF interferes with TGF-β signaling by inhibiting the Smad pathway, which is a major downstream signaling pathway of TGF-β. HGF can prevent the phosphorylation and nuclear translocation of Smad proteins, thereby inhibiting the transcription of pro-fibrotic genes. MSC-derived HGF can suppress the expression of alpha-smooth muscle actin (α-SMA), preventing the differentiation of fibroblasts into myofibroblasts [24]. In addition to their anti-fibrotic properties, MSCs are also able to suppress detrimental immune response in fibrotic lungs [25]. MSCs produce IL-1 receptor antagonist (IL-1Ra), which may attenuate pro-inflammatory effects of IL-1β on lung ECs, preventing a massive influx of circulating leukocytes in injured lungs [23,24,25]. Additionally, MSCs, in IL-10 and indoleamine 2,3-dioxygenase (IDO)-dependent manner, may promote the generation of anti-inflammatory phenotypes in lung dendritic cells (DCs), neutrophils and macrophages and may enhance expansion of immunosuppressive CD4+CD25+FoxP3-expressing T regulatory cells, enabling the creation of an immunosuppressive microenvironment in fibrotic lungs [23,24,25]. Finally, MSCs are a valuable source of pro-angiogenic factors (IL-6, vascular endothelial growth factor (VEGF), angiopoietin) which may crucially contribute to the re-vascularization and re-oxygenation of fibrotic lungs, resulting in the alleviation of hypoxemia-related signs and symptoms [25].

Despite the fact that MSCs have enormous therapeutic potential in pulmonology, several side effects caused by engrafted MSCs significantly limit their potential clinical use [26]. Despite the low expression of major histocompatibility class (MHC) II molecules, MSCs are not immune privileged cells, and the transplantation of allogeneic MSCs can trigger a detrimental immune response. The recipient’s immune system recognizes foreign MHC class I and II molecules on the engrafted MSCs, leading to rejection of the transplanted cells and the development of immune cell-driven inflammation. Another side effect of MSC transplantation is their unwanted differentiation. In certain cases, MSCs have been observed to spontaneously differentiate into chondrocytes and osteocytes, which compromises the structure, integrity and function of the tissue [26]. Several studies indicated that MSCs may facilitate the growth of pre-existing tumors [26,27]. This risk arises from the ability of MSCs to differentiate into various cell types, including those implicated in cancer development [26,27]. Moreover, pro-angiogenic factors derived from MSCs can potentially stimulate the growth of new blood vessels within the tumor microenvironment, facilitating the spread of malignant cells [27].

Since MSC-dependent beneficial effects in the treatment of lung fibrosis are mainly reliant on the biological activity of MSC-derived anti-fibrotic, immunoregulatory and angio-modulatory factors, injection of MSC-derived secretome is considered to be a novel approach for the treatment of pulmonary fibrosis, which may overcome all potential safety issues associated with the transplantation of MSCs [20]. The majority of MSC-sourced bioactive factors are contained within MSC-derived exosomes (MSC-Exos), nano-sized extracellular vesicles (EVs) which are abundantly present in the MSC-sourced secretome [28]. MSC-sourced EVs are, according to their size, classified into three types: exosomes (MSC-Exos; 30–150 nm), microvesicles (MSC-MVs; 150–1000 nm) and apoptotic bodies (MSC-APBs; larger than 1000 nm) [29]. Different types of MSC-derived EVs are synthesized by different processes. MSC-Exos are generated through the process of MSC-Exos’ biogenesis and MSC-MVs are released through pinching-off the plasma membrane via a direct budding process, while MSC-APBs are synthesized during apoptosis [29]. The process of MSC-Exos’ biogenesis begins as the endosomal pathway, where endosomes engulf extracellular material through endocytosis. The endosomes then mature into multivesicular bodies (MVBs) through the inward budding of the endosomal membrane [29,30]. This results in the formation of small vesicles known as intraluminal vesicles (ILVs) within the lumen of the MVBs. Once the MVBs are formed, they can either fuse with lysosomes for degradation or with the MSCs’ plasma membrane for the release of ILVs into the extracellular space as MSC-Exos [29]. Once released into the extracellular space, MSC-Exos can be taken up by target cells and participate in intercellular communication through the transfer of their cargo molecules [28]. The outer membrane of MSC-Exos is composed of phospholipids, cholesterol and glycolipids. Due to its small size and lipid envelope, MSC-Exos easily by-pass all biological barriers in the body and deliver their cargo directly to the target cells [28]. Additionally, MSC-Exos display adhesion molecules and chemokine receptors of their parental cells (CD9, CD63, CD81, CD44, CCR2), enabling rapid recruitment of MSC-Exos to the site of inflammation and injury [28]. MSC-Exos contain a variety of bioactive molecules, including proteins (growth factors, immunoregulatory molecules, cytokines, chemokines), lipids and nucleic acids (messenger RNA (mRNA) and microRNAs (miRNAs)) which affect the viability, proliferation, phenotype and function of parenchymal and immune cells in injured and inflamed tissues [25]. The results obtained in recently published experimental studies demonstrated beneficial effects of MSC-Exos in the treatment of lung fibrosis, suggesting their potential therapeutic use in clinical settings [21,28,31]. The therapeutic potential of MSC-Exos was reliant upon the biological effects of MSC-sourced growth factors, immunoregulatory proteins and anti-apoptotic miRNAs which were able to (i) improve the survival and viability of alveolar epithelial cells, (ii) suppress detrimental immune response, (iii) attenuate on-going inflammation, (iv) inhibit activation of fibroblasts, (v) prevent generation of miofibroblasts and (vi) reduce extensive collagen production and accumulation in inflamed lungs, crucially contributing to the MSC-dependent attenuation of pulmonary fibrosis [21,28,31]. Accordingly, in this review article, we emphasized the current understanding of the molecular and cellular processes that contributed to the anti-fibrotic, immunosuppressive and angiomodulatory effects of MSC-Exos in the treatment of pulmonary fibrosis. An extensive literature review was carried out in January 2024 across several databases (MEDLINE, EMBASE, Google Scholar), from 2000 to present. The keywords used in the selection were: “mesenchymal stem cells”, “exosomes”, “lung fibrosis”, “lung inflammation”, “miRNAs”, “signaling pathways”, “fibroblasts”, “myofibroblasts”, “immunomodulation”, “tissue repair and regeneration”. All journals were considered and the initial search retrieved 148 articles. The abstracts of all these articles were subsequently reviewed by two of the authors (CRH and VV) independently to check their relevance to the subject of this manuscript. Eligible studies had to delineate molecular and cellular mechanisms responsible for the MSC-Exos-based modulation of lung fibrosis and their findings were analyzed in this review.

## 3. MSC-Exo-Dependent Suppression of Immune Cells in Fibrotic Lungs

A large number of recently published experimental studies provided evidence about the therapeutic potential of MSC-Exos in the treatment of lung fibrosis [28,31,32,33,34]. MSC-Exos increased survival of alveolar epithelial cells, reduced activation of fibroblasts, prevented their differentiation in myofibroblasts, inhibited lung inflammation and suppressed the pro-fibrotic effects of immune cell-derived cytokines (Table 1) [28,31,32,33,34].

**Figure 1:**
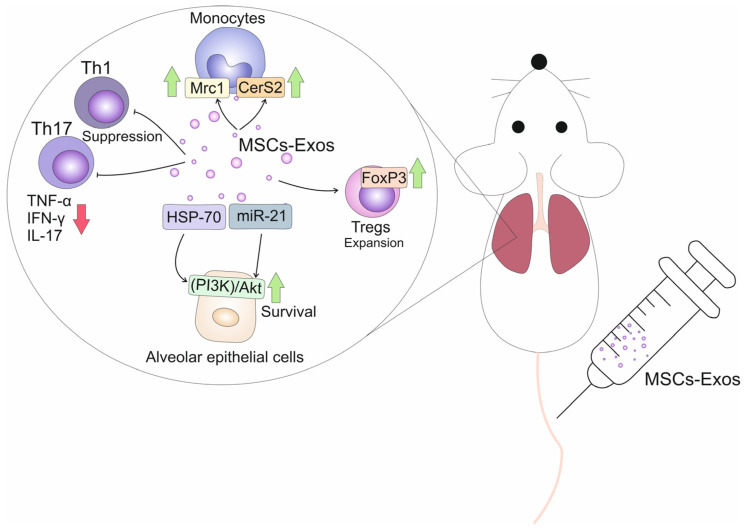
Molecular mechanisms responsible for MSC-Exo-dependent suppression of immune cells in fibrotic lungs. MSC-Exos altered cellular-make up of fibrotic lungs by affecting survival, phenotype and function of lung-infiltrated immune cells. In inflamed and fibrotic lungs, MSC-Exos reduced production of inflammatory cytokines (TNF-α, IFN-γ, IL-17) in Th1 and Th17 lymphocytes (arrow down) and increased the synthesis of immunoregulatory proteins (Mrc1 and CerS2) in monocytes (arrow up), ameliorating their immunosuppressive properties. By delivering anti-apoptotic miR-21 and HSP-70, MSC-Exos improved survival and viability of alveolar epithelial cells, further contributing to the attenuation of lung fibrosis.

By delivering MSC-sourced immunoregulatory factors in lung-infiltrated monocytes and T cells, MSC-Exos modulate their function, alleviating on-going inflammation and fibrosis (Figure 1) [28,31]. Mansouri and colleagues used a mice model of bleomycin-induced pulmonary fibrosis to assess the effects of MSC-Exos on phenotypes of lung monocytes [33]. A single intravenous injection of MSC-Exos managed to prevent and revert bleomycin-induced pulmonary fibrosis in experimental animals. MSC-Exos (8.6 × 10^8^ particles in 200 μL) which were intravenously given concurrently with bleomycin (on day 0) prevented apoptosis of alveolar epithelial cells, improved the Ashcroft score and inhibited the synthesis and deposition of collagen in mice lungs [33]. By delivering anti-apoptotic miR-21 and heat shock protein (HSP)-70, which have cytoprotective properties, MSC-Exos down-regulated expression of pro-apoptotic genes and activated the phosphatidylinositol-3-kinase (PI3K)/Akt-driven anti-apoptotic signaling pathway in alveolar epithelial cells, improving their survival and viability [33]. The alveolar epithelial cells play a crucial role in the regulation of lung homeostasis and repair. When these cells are healthy and able to function properly, they can effectively remove debris and toxins from the alveoli, as well as produce surfactants to maintain the surface tension of the alveoli [37]. By promoting the survival of alveolar epithelial cells, MSC-Exo-sourced miR-21 maintains the structural integrity of the lungs, reduces inflammation and improves overall lung function, ultimately attenuating the development of lung fibrosis [33]. In addition, alveolar epithelial cells play a crucial role in maintaining the barrier function of the lungs. These specialized cells are responsible for forming a physical barrier that separates the air in the alveoli from the surrounding tissue and blood vessels [38]. Therefore, by suppressing apoptosis of alveolar epithelial cells, MSC-Exo-miR-21 contributes to the maintenance of the lung’s barrier function, which is essential for preventing the infiltration of inflammatory cells and further damage to the lung tissue [33].

The monocyte-attracting chemokine C–C motif chemokine ligand 2 (CCL2) is mainly responsible for the enhanced influx of circulating monocytes in inflamed lungs [39]. MSC-Exos highly express CCL2-specific receptor (CCR2) which binds to CCL2 on the surface of monocytes/macrophages to inhibit their influx in fibrotic lungs [40]. Additionally, by interfering with the CCL2-CCR2 signaling cascade, MSC-Exos reprogram “classical”, inflammatory monocytes (CD45+CD11b+MHCII–CD64–CCR-2+Ly6Chi) into “non-classical”, anti-inflammatory cells (CD45+CD11b+MHCII–CD64–CCR-2-Ly6Clo), attenuate the production of inflammatory and pro-fibrotic cytokines (TNF-α, IL-1β, IL-6, TGF-β) and promote the synthesis of immunosuppressive IL-10 [33,40]. MSC-Exos significantly reduced activation of mitogen-activated protein kinases (MAPKs) and modulated the synthesis of serpin proteins, which play a crucial role in regulating protease activity in monocytes, maintaining the balance between monocyte-driven lung inflammation and tissue remodeling [33]. Additionally, MSC-Exos increased the synthesis of immunoregulatory proteins (mannose receptor C-type 1 (Mrc1) and ceramide synthase 2 (CerS2)) in lung-infiltrated monocytes [33]. Mrc1 is a type I transmembrane protein expressed on the surface of various immune cells, including alveolar macrophages [41]. It acts as a pattern recognition receptor, recognizing and binding to microbial pathogens that invaded lungs [2,41]. Mrc1 has been shown to have immunoregulatory functions by promoting anti-inflammatory and tissue repair responses. Mrc1 engagement by its ligands, such as mannose-containing carbohydrates and extracellular matrix components, triggers downstream signaling pathways that lead to the production of anti-inflammatory cytokine IL-10 and the suppression of pro-inflammatory cytokines (TNF-α and IL-1β), alleviating on-going lung inflammation [2,41]. Mrc1 has been also associated with tissue repair processes. It can promote the clearance of apoptotic cells, cellular debris and extracellular matrix components, which are crucial for tissue remodeling and the resolution of lung inflammation [41]. CerS2 is an enzyme involved in the synthesis of ceramides, which are bioactive lipids with diverse functions in lung inflammation and fibrosis. CerS2-generated ceramides inhibit the production of pro-inflammatory cytokines (IL-1β and IL-6) in alveolar macrophages, attenuate the production of extracellular matrix components and inhibit the differentiation of fibroblasts, importantly contributing to the resolution of fibrosis [2,41]. In line with these findings, Mansouri and colleagues demonstrated that both early injected MSC-Exos (day 7) and late-rescue administered MSC-Exos (infused on day 21) managed to significantly reduce the bleomycin-induced elevation in collagen content in fibrotic mice lungs, indicating the therapeutic potential of MSC-Exos in the treatment of pulmonary fibrosis [33].

Th17 cells play an important pathogenic role in the development and progression of pulmonary fibrosis [42]. The cross-talk between Th17 lymphocytes and neutrophils aggravates macrophage-driven immune responses in inflamed lungs [4]. Th17 cell-sourced IL-17 activates neutrophils, to release proteases and reactive oxygen species (ROS) which contribute to the destruction of lung tissue and the activation of fibroblasts [2]. Th17 cell-sourced inflammatory cytokines induce activation of fibroblasts and promote their differentiation into myofibroblasts [43]. Th17-derived IL-17 and IL-22 stimulate fibroblasts to produce extracellular matrix proteins (collagen, fibronectin and elastin), leading to the accumulation of scar tissue in the lungs [43]. Additionally, Th17 cells can inhibit the immunosuppressive activity of CD4+FoxP3-expressing Tregs, favoring the progression of on-going lung inflammation and fibrosis [4]. Chronic inflammatory response in the lungs results in the continuous antigen-dependent priming of T cell receptors, resulting in the phosphorylation of protein kinase B (PKB/Akt) and mammalian target of rapamycin (mTOR) in resting Tregs [44,45]. The activation of Akt/mTOR pathways alters the immunoregulatory phenotype of Tregs and induces their reprogramming into a pro-inflammatory Th17-like phenotype [45]. MSC-Exos deliver MSC-derived IDO, an enzyme which metabolizes and attenuates the concentration of tryptophan (TRP) in inflamed microenvironments [28]. Low levels of TRP induce activation of general control nonderepressible 2 (GCN2) kinase, which inhibits Akt/mTOR2 signaling in Tregs [45]. Accordingly, MSC-Exos, in an IDO-dependent manner, induce the generation of Foxp3-expressing Tregs and prevent their transdifferentiation in inflammatory Th17 cells in inflamed lungs and in an MSC-Exo-dependent manner suppress T cell-driven lung inflammation and fibrosis [28]. In line with these findings are results recently obtained by Lai and colleagues, who demonstrated beneficial effects of bone marrow (BM)-derived MSC-Exos in the attenuation of lung fibrosis [34]. BM-MSC-Exos suppressed Th17 cell-driven lung fibrosis and induced expansion of Tregs in fibrotic lungs of experimental mice, resulting in the alleviation of pulmonary fibrosis [34]. In vitro, BM-MSCs-Exos down-regulated the expression of genes that regulate Th17 differentiation and favored activity of Treg-related FoxP3 transcriptional factor, enabling the expansion of Tregs in the population of activated human peripheral blood mononuclear cells (pbMNCs) [34]. Similarly, amniotic fluid-derived MSC-Exos contained within a derived-multiple allogeneic proteins paracrine signaling (d-MAPPS) inhalation solution suppressed Th17 cell-driven lung inflammation in experimental animals, inhibited the production of pro-inflammatory and pro-fibrotic cytokines (IL-17, TNF-α, IL-1β) and enhanced the secretion of anti-inflammatory IL-10 in human pbMNCs [46,47], confirming the therapeutic potential of MSC-Exos in the modulation of T cell-dependent pulmonary inflammation and fibrosis.

## 4. MSC-Exo-Based Attenuation of Pulmonary Fibrosis Is Based on the Modulation of Wnt-Driven Signaling Pathways

Silica dust-induced pulmonary fibrosis is an irreversible, fibroproliferative lung disease and currently, there are no effective treatments available for this disease [48]. Zhang and colleagues demonstrated that systemic infusion of bone marrow (BM)-derived MSCs-Exos attenuated silica-induced pulmonary fibrosis [35]. BM-MSC-Exos suppressed production of pro-fibrotic TGF-β1 in lung-infiltrated immune cells and prevented the progression of EMT in inflamed lungs [35]. MSCs-Exos increased the expression of epithelial marker proteins (E-cadherin, cytokeratin 19) and reduced the expression of fibrosis marker protein α-SMA after exposure to silica suspension [35].

Beneficial effects of MSCs-Exos were reliant on the inhibition of the Wnt/β-catenin pathway in fibrotic lungs [35]. In normal lung tissue, the Wnt/β-catenin pathway is tightly regulated, maintaining a balance between the inactive and active forms of β-catenin [49]. In the absence of Wnt ligands, β-catenin is phosphorylated by a destruction complex consisting of adenomatous polyposis coli (APC), Axin, glycogen synthase kinase-3β (GSK-3β) and casein kinase 1α (CK1α). Phosphorylated β-catenin is targeted for degradation by the proteasome, resulting in low levels of cytoplasmic β-catenin [49]. In pulmonary fibrosis, aberrant activation of the Wnt/β-catenin pathway occurs, leading to the accumulation and nuclear translocation of β-catenin. This activation is triggered by TGF-β and ROS released by activated lung-infiltrated inflammatory immune cells [4,49]. The accumulation of β-catenin in the nucleus allows it to bind to T cell factor/lymphoid enhancer factor (TCF/LEF) transcription factor, leading to the transcriptional activation of target genes involved in fibrosis [49]. The activation of the Wnt/β-catenin pathway induces an increased production of pro-inflammatory cytokines and chemokines, leading to the recruitment and activation of immune cells. It also stimulates the proliferation and activation of fibroblasts, resulting in the increased production and deposition of collagen and fibronectin, leading to tissue remodeling and scarring [49]. Zhang and colleagues revealed that MSC-Exos attenuated the progression of silica-induced pulmonary fibrosis by suppressing expression of GSK3β and β-catenin which inhibited Wnt/β-catenin-driven fibrosis in the lungs [35].

Intravenously injected MSC-Exos attenuated pulmonary vascular remodeling and lung fibrosis by down-regulating the gene expression of β-catenin, cyclin D1 and TGF-β1 and by enhancing expression of Wnt5a and BMPR2 (Bone Morphogenetic Protein Receptor Type 2) [36]. Wnt5a-drven signaling is distinct from the canonical Wnt/β-catenin pathway and is known as the non-canonical Wnt pathway [50]. In normal lung tissue, Wnt5a is expressed at low levels, but its expression is up-regulated in response to pro-fibrotic TGF-β. Wnt5a has been reported to have antifibrotic effects by inhibiting the activation and differentiation of fibroblasts into myofibroblasts [50]. Wnt5a can inhibit the TGF-β-induced expression of collagen and TGF-β-mediated EMT in inflamed lungs [50]. BMPR2 is a receptor for BMP ligands and is expressed in endothelial cells, smooth muscle cells and fibroblasts [51]. Mutations in the BMPR2 gene have been associated with pulmonary arterial hypertension and fibrosis. In lung fibrosis, reduced expression or dysfunction of BMPR2 has been observed [51]. This down-regulation of BMPR2 is associated with increased fibroblast activation, excessive extracellular matrix (ECM) deposition and tissue remodeling [51]. Zhang and colleagues showed that MSC-Exos increased Wnt5a/BMP2-driven signaling and suppressed Wnt/β-catenin-dependent collagen deposition and EMT in inflamed and fibrotic lungs, which resulted in the attenuation of pulmonary fibrosis [36].

## 5. The Role of MSC-Exo-Derived miRNAs in the Attenuation of Lung Fibrosis

MSC-Exos contain a large number of MSC-sourced miRNAs, which are small, around 22 nucleotides in length, non-coding RNA molecules that play a crucial role in post-transcriptional gene regulation [28]. MiRNAs present in MSC-Exos can be transferred to recipient cells, where they can regulate gene expression and influence cellular functions [52]. Once taken up by recipient cells, the miRNAs can bind to target mRNAs, leading to mRNA degradation or translational repression. This, in turn, can influence the expression of specific genes and impact cellular phenotype and function [28,52]. MSC-derived miRNAs are involved in various biological processes, including development, cell differentiation, metabolism and immune response. The specific miRNAs present in MSC-Exos can vary depending on various factors, including the source of the MSCs, the culture conditions and the cellular environment [52].

**Figure 2:**
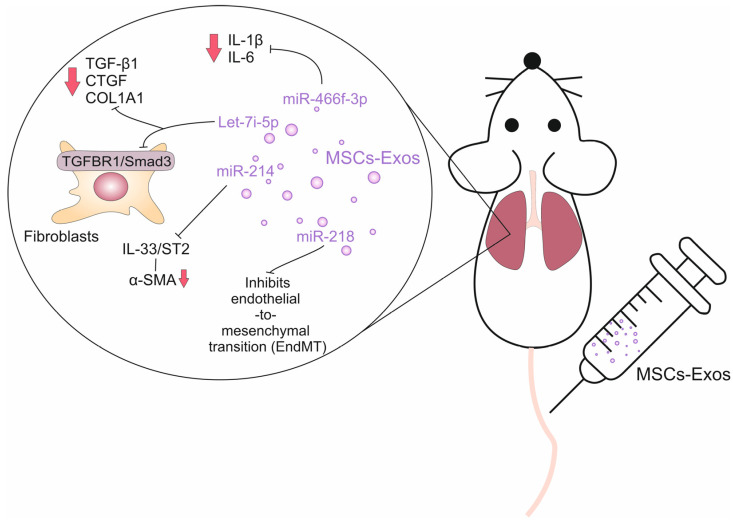
Molecular mechanisms responsible for the beneficial effects of MSC-Exo-derived miRNAs in the alleviation of pulmonary fibrosis. MSC-Exo-sourced miRNAs (Let-7i-5p, miR-218, miR-214, miR-466f-3p) inhibited production (arrows down) of inflammatory cytokines in lung-infiltrated leukocytes, suppressed detrimental immune response, prevented differentiation of fibroblasts in myofibroblasts and reduced collagen production and extracellular matrix deposition in inflamed lungs, which resulted in the attenuation of pulmonary fibrosis.

The transfer of miRNAs through MSC-Exos provides a mechanism for cell-to-cell communication and a potential therapeutic avenue for harnessing the regenerative and immunomodulatory properties of MSCs [52]. By delivering specific miRNAs to target cells, MSC-Exos hold potential for modulating gene expression and influencing cellular functions in pulmonary fibrosis (Figure 2) [53,54,55,56,57]. Several recently published studies have shown that MSC-Exo-delivered miRNAs modulated detrimental immune response in fibrotic lungs, modulated EMT and regulated collagen production in fibroblasts (Table 2) [53,54,55,56,57].

Xu and colleagues used a mice model of silica-induced lung fibrosis to evaluate the therapeutic potential of MSC-Exos in the treatment of this life-threatening condition [53]. They showed that systemic, intravenous injection of human umbilical cord MSC-Exos completely suppressed the development and progression of pulmonary fibrosis in experimental animals by down-regulating gene expression of collagen type I alpha 1 (COL1A1) and fibronectin (FN) [53]. MSC-derived Let-7i-5p was mainly responsible for the beneficial effects of MSC-Exos [54]. MSC-Exo-sourced Let-7i-5p inhibited the TGFBR1/Smad3 signaling pathway in lung fibroblasts, preventing their activation and consequent differentiation in myofibroblasts [54]. Mechanistically, MSC-Exo-sourced Let-7i-5p suppressed the expression of TGF-β1, connective tissue growth factor (CTGF) and COL1A1 genes in fibroblasts, preventing excessive collagen production and extracellular matrix deposition in inflamed lungs. Also, MSC-Exo-sourced Let-7i-5p induced apoptosis and senescence in fibroblasts and myofibroblasts, limiting their fibrogenic potential [54]. Additionally, MSC-Exo-derived Let-7i-5p inhibited EMT by targeting Snail and Twist proteins. By inhibiting EMT, Let-7i-5p helps maintain the integrity of the epithelial barrier and prevents the infiltration of circulating leukocytes into the inflamed lungs [54]. In lung-infiltrated inflammatory monocytes, MSC-Exo-sourced Let-7i-5p inhibited the production of pro-inflammatory cytokines (IL-6 and TNF-α), preventing inflammation-driven tissue damage and fibrosis [54].

Zhao and colleagues showed that MSC-Exo-sourced miR-218 attenuated bleomycin-induced pulmonary fibrosis by inhibiting endothelial-to-mesenchymal transition (EndMT) in the lungs [55]. EndMT is a biological process in which endothelial cells undergo a transformation into mesenchymal cells [58]. This transition involves a loss of endothelial characteristics and acquisition of mesenchymal features, such as increased migratory and invasive properties. During EndMT, endothelial cells lose their tight junctions and acquire a more mesenchymal-like phenotype. They upregulate the expression of mesenchymal markers, such as α-SMA and fibroblast-specific protein 1 (FSP1) [58]. These transformed cells can then migrate into the surrounding tissue and contribute to the fibrotic response by promoting the production of ECM proteins and by activating fibroblasts [58]. Several signaling pathways have been implicated in driving EndMT in lung fibrosis, including TGF-β, BMP2 and Methyl-CpG Binding Protein 2 (MeCP2)-driven signaling [58]. These pathways promote the activation of transcription factors, such as Snail, Twist, and Slug, which are known to regulate the processes of EndMT and pulmonary fibrosis [51]. In bleomycin-induced pulmonary fibrosis, pathways elicited by TGFβ and BMP2 follow an inverse course [51]. Continuous activation of TGFβ and repression of BMP2-driven signaling can lead to the progression and aggravation of lung fibrosis [51]. MeCP2 is an activator of α-SMA expression in human lung myofibroblasts [59]. MeCP2 also contributes to the development of lung fibrosis through the suppression of the BMP2 pathway. Overexpressed MeCP2 aggravated EndMT and caused increased CpG islands methylation at the BMP2 promoter, which led to BMP2 post-transcriptional gene silence [51,59]. In an animal model of bleomycin-induced pulmonary fibrosis, Zhao and colleagues showed that human umbilical cord-derived MSC-Exos elevated miR-218 expression and restored endothelial properties weakened by TGF-β and MeCP2-dependent suppression of BMP2 [55]. Knockdown of miR-218 in MSCs partially abrogated the beneficial effects of MSC-Exos, while transfection of miR-218 and its overexpression in MSC-Exos resulted in increased BMP2 expression, which led to the attenuation of EndMT and alleviation of pulmonary fibrosis [55].

Xie and colleagues recently showed that BM-MSC-Exo-sourced miR-214 inhibited progression of bleomycin-induced pulmonary fibrosis by suppressing the IL-33/ST2 axis in fibrotic lungs [56]. IL-33 is an immunomodulatory cytokine and alarmin which is released from injured endothelial and alveolar epithelial cells during the progression of detrimental inflammatory response in the lungs [60]. IL-33 promotes lung fibrosis by recruiting and activating lung-infiltrated inflammatory immune cells (monocytes, mast cells, Th2, Th17 lymphocytes) by inducing enhanced secretion of pro-fibrotic cytokines (TGF-β and IL-13), by activating fibroblasts causing them to differentiate into myofibroblasts and by promoting EMT [60]. Xie and colleagues revealed that BM-MSC-Exo-sourced miR-214 targeted IL-33 and blocked the IL-33/ST2 axis in fibroblasts and lung-infiltrated immune cells, which reduced collagen fiber accumulation and α-SMA production, resulting in the attenuation of bleomycin-induced pulmonary fibrosis in experimental mice [56].

In line with these findings are recently reported results by Li and colleagues, who demonstrated that MSC-Exo-derived miR-466f-3p attenuated radiation-induced lung fibrosis by suppressing EMT in irradiated lungs [57]. MSC-Exo-sourced miR-466f-3p reduced the production of inflammatory cytokines (IL-1β and IL-6) in lung-infiltrated immune cells and inhibited collagen production in lung fibroblasts [57]. Mechanistically, MSC-Exo-derived miR-466f-3p inhibited the synthesis of the SNAIL protein by suppressing activation of the AKT/GSK3β signaling pathway in a c-MET-dependent manner [57]. SNAIL is a transcription factor that plays a crucial role in the development of EMT in the lungs [61]. The activation of SNAIL and subsequent EMT in lung fibrosis leads to several pathological changes. It causes the disruption of the epithelial barrier, allowing the infiltration of inflammatory cells and fibrogenic mediators into the lung tissue [61]. SNAIL also promotes the activation of fibroblasts, enabling excessive ECM deposition in fibrotic lungs. SNAIL-induced EMT also enhances fibroblast migration and, contractility, contributes to the formation of fibrotic foci [61]. SNAIL is up-regulated in response to pro-fibrotic TGF-β. Once activated, SNAIL represses the expression of E-cadherin, an epithelial marker, and promotes the expression of mesenchymal markers, such as N-cadherin, vimentin and α-SMA. The activation of SNAIL in irradiated lungs is regulated by AKT and GSK3β kinases. Upon the inhibition of AKT, radiation-induced repression of GSK-3β is released, which correlates with the down-regulation of SNAIL in inflamed and fibrotic lungs [61]. As demonstrated by Li and colleagues, by modulating the AKT/GSK3β signaling pathway in alveolar epithelial cells, MSC-Exo-derived miR-466f-3p suppressed SNAIL activity, prevented EMT and attenuated pulmonary fibrosis in irradiated mice [57].

## 6. Conclusions and Future Perspectives

In recent years, MSC-Exos have gained significant attention as potential vehicles for the delivery of immunomodulatory agents and trophic factors [28]. The use of MSC-Exos for delivering therapeutic agents offers several advantages. Firstly, MSC-Exos are naturally occurring nanoparticles that have intrinsic properties for efficient drug delivery. They possess a lipid bilayer membrane that protects the encapsulated cargo from degradation and clearance. This membrane also allows for easy cellular uptake, facilitating the targeted delivery of therapeutic agents to fibrotic sites. Secondly, MSC-Exos have inherent immunomodulatory and anti-inflammatory properties. These properties can help dampen the immune response and create a favorable environment for tissue repair and regeneration [28].

In terms of therapeutic applications in pulmonary fibrosis, MSC-Exos can be loaded with various anti-fibrotic agents, such as small molecules, siRNAs, miRNAs or proteins. The encapsulation of these agents within MSC-Exos enhances their stability, bioavailability and targeted delivery within fibrotic lungs [28]. Based on these observations, Cai and colleagues loaded adipose tissue-derived MSC-Exos with Nintedanib (Nin), a tyrosine kinase inhibitor used to treat idiopathic pulmonary fibrosis [62]. MSC-Exo-Nin significantly attenuated bleomycin-induced pulmonary fibrosis by suppressing ROS and TGF-β-driven lung injury, inflammation and fibrosis. MSC-Exo-Nin prevented the development and progression of EMT through the down-regulation of the TGF-β/Smad signaling pathway in fibrotic lungs [62]. Importantly, lung fibrosis was more efficiently alleviated in MSC-Exos-Nin-treated than in Nin-only-treated animals, confirming the anti-fibrotic properties of MSC-Exos and their therapeutic potential in the treatment of pulmonary fibrosis [62].

Although numerous pre-clinical studies have demonstrated the therapeutic potential of MSC-Exos in the treatment of pulmonary fibrosis, it is important to acknowledge that there are several challenges that currently hinder their clinical implementation [63,64]. Standardization of protocols for the isolation and characterization of MSC-Exos is lacking, leading to variations in purity, size and content [63]. It is crucial to establish standardized procedures to ensure consistent and reproducible results. Additionally, the production of MSC-Exos in large quantities for clinical use is challenging, as current methods are time-consuming, expensive and yield low quantities of EVs [64]. Developing scalable and cost-effective production methods is necessary to meet the demands of clinical applications [63,64]. Additionally, it should be noted that MSC-Exos are sensitive to environmental conditions such as temperature, freeze–thaw cycles and storage duration [64]. Maintaining the stability and integrity of MSC-Exos during storage and transportation is critical for their clinical application [63]. Identifying specific markers or cargo profiles associated with therapeutic efficacy and developing appropriate storage conditions, such as cryopreservation, are necessary to preserve the functional properties and therapeutic efficacy of MSC-Exos [63,64].

MSCs derived from different tissues exhibit variations in their phenotypic and functional properties due to the influence of the tissue microenvironment [65]. This heterogeneity of MSCs can result in variations in the content of MSC-Exos isolated from different tissue sources. Also, the heterogeneity of MSC-Exos poses challenges in defining a specific set of functional characteristics for clinical use and can lead to inconsistent therapeutic effects [65]. Therefore, considering the fact that MSC-Exos contain numerous bioactive proteins and miRNAs, the safety profile of each MSC-Exos-based therapeutic agent needs to be thoroughly evaluated in clinical trials before they can be offered as new therapeutic options for the treatment of lung fibrosis.

## Figures and Tables

**Table 1:** Molecular mechanisms responsible for MSC-Exo-dependent effects on alveolar epithelial cells, fibroblasts and immune cells in inflamed and fibrotic lungs.

Target Cell(s)	Mechanism(s) of Action	Biological Effect(s)	Ref. No.
Alveolar epithelial cells	HSP-70-dependent activation of PI3K/Akt- signaling pathway;	Improved survival and viability of alveolar epithelial cells	[33]
Monocytes/ macrophages	CCR2-based suppression of CCL2-driven signaling pathway; Reprogramming of “classical”, inflammatory M1 macrophages into anti-inflammatory, immunosuppressive M2 macrophages	Attenuated production of inflammatory and pro-fibrotic cytokines (TNF-α, IL-1β, IL-6, TGF-β); Increased synthesis of immunosuppressive IL-10	[33]
Tregs; Th17 lymphocytes	IDO-dependent activation of GCN2 kinase and inhibition of Akt/mTOR2-driven signaling	Increased expansion of Tregs; Prevented differentiation of Tregs into Th17 cells; Suppressed production of IL-17	[34]
Alveolar epithelial cells; lung-infiltrated immune cells	Inhibition of Wnt/β-catenin pathway	Increased expression of E-cadherin and cytokeratin 19 and reduced expression of α-SMA; Attenuated production of TGF-β	[35]
Fibroblasts	Down-regulated gene expression of β-catenin, cyclin D1 and TGF-β1; Activation of Wnt5a/BMP2-signaling pathway	Reduced collagen production and extracellular matrix deposition in inflamed lungs;	[36]

**Table 2:** Therapeutic potential of MSC-derived miRNAs in pulmonary fibrosis.

MSC-Derived miRNA	Animal Model	Target Cell(s)	Mechanism(s) of Action	Biological Effect(s)	Ref. No.
MiR-21	Mice model of bleomycin-induced pulmonary fibrosis	Alveolar epithelial cells; Monocytes/ macrophages	Reduced synthesis of pro-apoptotic proteins; Increased synthesis of immunoregulatory proteins (Mrc1 and CerS2)	Improved viability of alveolar epithelial cells; Reduced production of TNF-α and IL-1β; Increased synthesis of IL-10	[33]
Let-7i-5p	Mice model of silica-induced lung fibrosis	Lung fibroblasts; Alveolar epithelial cells; Lung-infiltrated monocytes	Inhibition of TGFBR1/Smad3 signaling pathway; Inhibition of epithelial-to-mesenchymal transition; Suppressed production of TNF-α and IL-1β	Reduced collagen production and extracellular matrix deposition in inflamed lungs; Preserved integrity of the epithelial barrier and reduced influx of circulating immune cells in the fibrotic lungs	[54]
MiR-218	Mice model of bleomycin-induced pulmonary fibrosis	Endothelial cells	Increased BMP2 expression; Inhibition of endothelial-to-mesenchymal transition	Reduced extravasation of immune cells from the blood vessels n inflamed lungs	[55]
MiR-214	Mice model of bleomycin-induced pulmonary fibrosis	Lung fibroblasts; Lung-infiltrated immune cells	Inhibition of IL-33/ST2 signaling pathway	Reduced collagen and α-SMA production; Attenuated production of pro-fibrotic TGF-β	[56]
miR-466f-3p	Radiation-induced lung fibrosis	Lung fibroblasts; Lung-infiltrated immune cells; Alveolar epithelial cells	Suppressed activation of AKT/GSK3β signaling pathway; Reduced synthesis of SNAIL protein	Reduced collagen production; Attenuated production of TNF-α and IL-1β; Inhibition of epithelial-to-mesenchymal transition	[57]

## Data Availability

The data that are discussed in this article are presented in the cited studies.
